# Supplementary material for: Cryo-EM study of start codon selection during archaeal translation initiation
Source: Nat Commun. 2016 Nov 7;7:13366. doi: 10.1038/ncomms13366 (PMC5103072; doi:10.1038/ncomms13366)
Supplement: Supplementary Information — Supplementary Figures 1 - 8, Supplementary Table 1 and Supplementary References [file ncomms13366-s1.pdf]

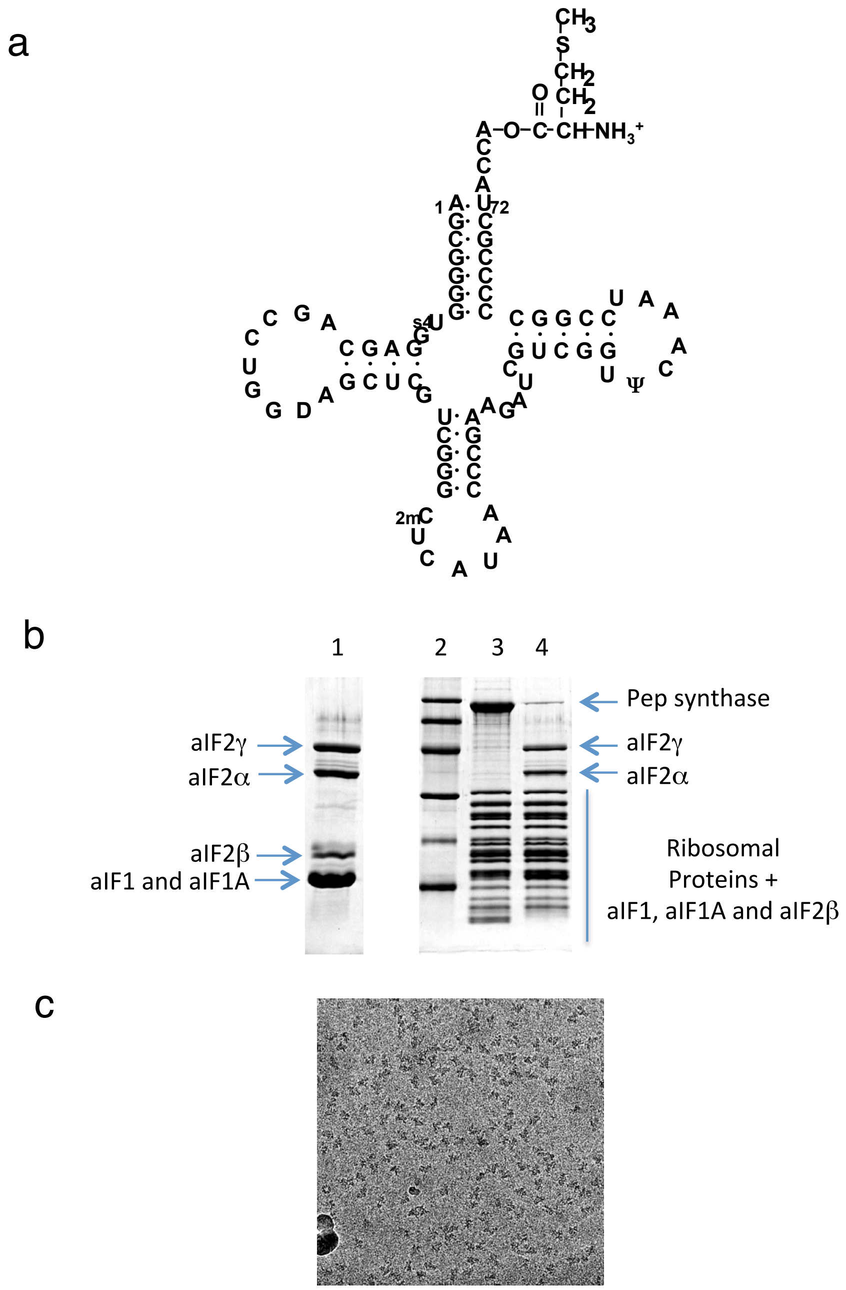

Supplementary Figure 1: Assembly of IC.
(a) Cloverleaf representation *E. coli* initiator $tRNA_f^{Met}$ carrying the mutation C_1 A_{72} into A_1 U_{72}.
(b) SDS-PAGE analysis of purification steps of the IC.
Lane 1: Mixture of purified Pa-aIF2, Pa-aIF1 and Pa-aIF1A. Note that N-terminally tagged versions of Pa-aIF1, Pa-aIF2α and Pa-aIF2β were used. aIF1 and aIF1A co-migrate. Lane 2: molecular weight marker (LMW, 97, 64, 43, 30, 20, 14 kDa, GE-Healthcare). Lane 3: 30S subunit from *P. abyssi* purified as described in Methods. The band corresponding to phosphoenol pyruvate synthase (Pep synthase) is indicated by an arrow. Lane 4: IC complex after affinity column purification and concentration. Bands corresponding to aIF2γ and to aIF2α are indicated. Bands corresponding to aIF1, aIF1A and aIF2β co-migrate with ribosomal proteins. The presence of tRNA was verified using staining with Ethidium Bromide.
(c) Cryo-EM image of IC collected on an FEI Cs-corrected Titan Krios microscope (-2.3 µm defocus) operated at 300kV using a 4k x 4k FEI Falcon II camera (Netherlands Centre for Electron Nanoscopy, Leiden).

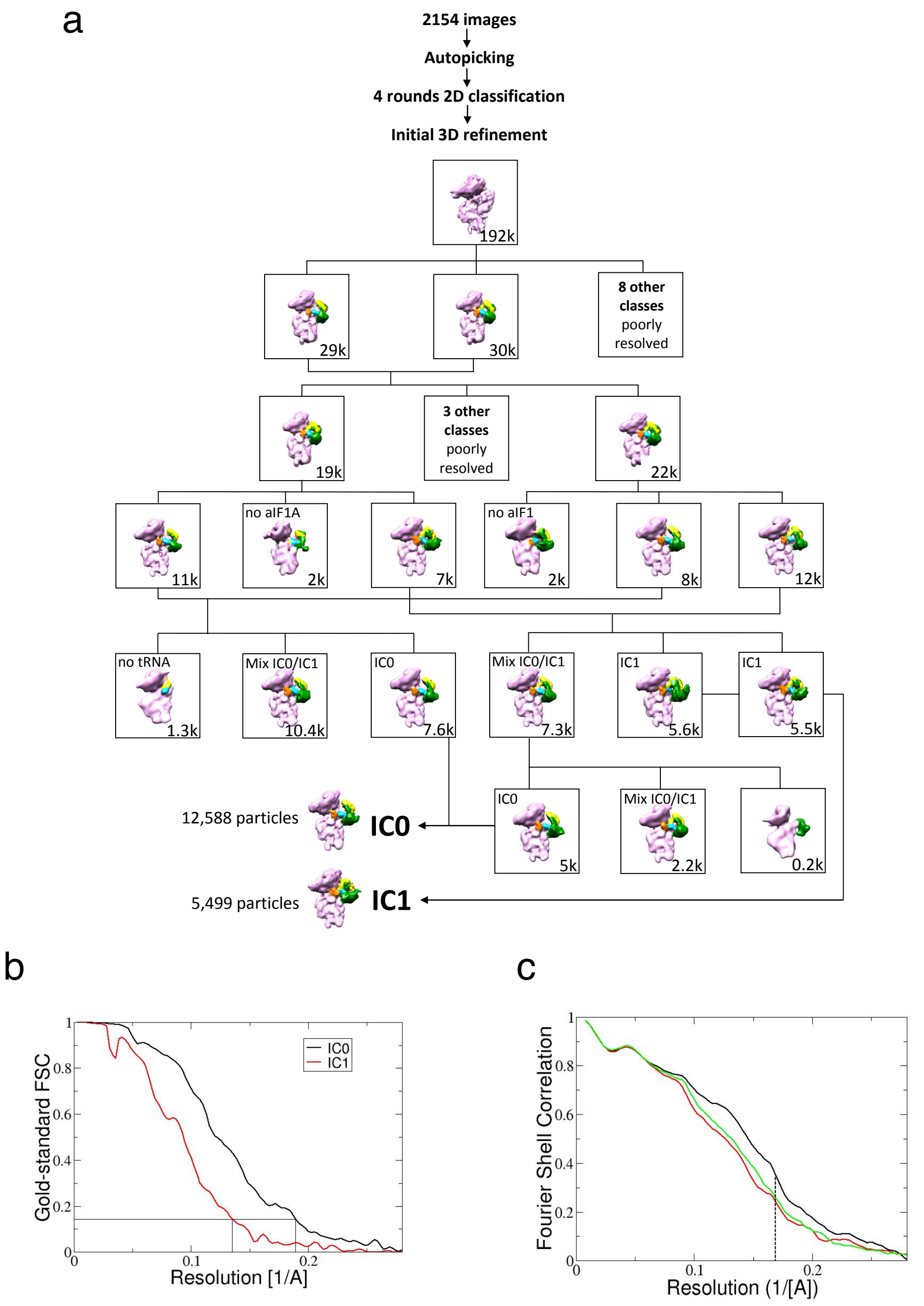

Supplementary Figure 2: Image processing and structure refinement.
(a) Maximum-likelihood 3D classification scheme (see Methods). The initiation factors bound to the 30S subunit (pink) are colored as follows: aIF2 green, aIF1A orange, aIF1 cyan and tRNA yellow.
(b) Gold-standard Fourier Shell Correlation (FSC) curves for post-processed maps. The resolutions of IC0-P_{REMOTE} (in black) and IC1-P_{IN} (in red) are 5.34 Å, and 7.5 Å respectively, using the 0.143 FSC cut-off criterion.
(c) Analysis of overfitting by cross-validation of the IC0-P_{REMOTE} complex model.
FSC_{work} curve (green) corresponds to the refined model versus the half-map it was refined against. The FSC_{test} curve (red) is calculated between the refined model and the other half-map. The black curve shows the FSC between the refined model and the full map used for refinement. The dashed line represents the highest resolution (5.8 Å) used in refinement.
The figures were drawn with Chimera[1] and Pymol[2].

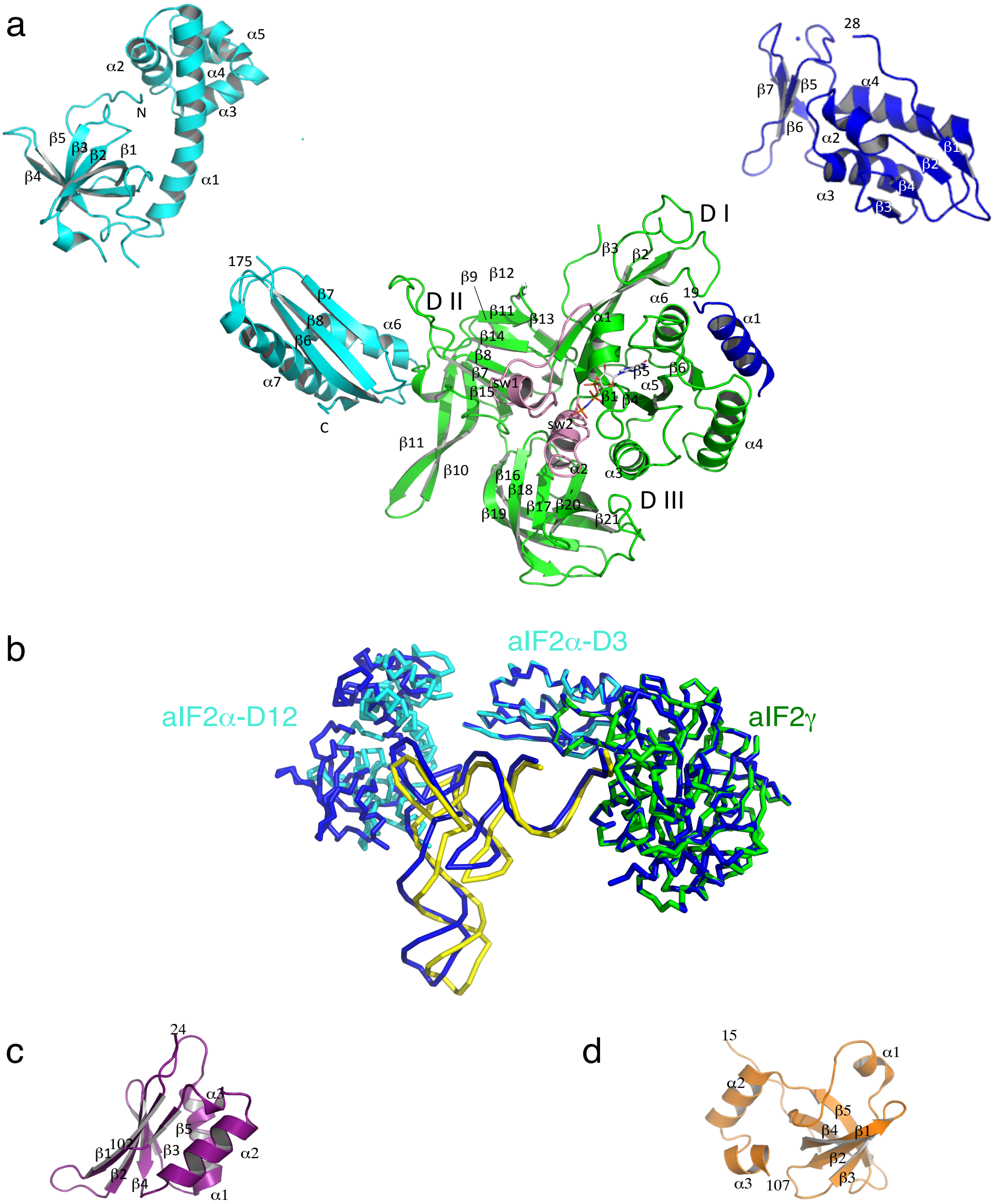

Supplementary Figure 3: Structures of initiation factors used for model building.

(a) aIF2 is composed of a core domain (aIF2γ-aIF2α–D3-Nterminal helix of aIF2β) onto which two mobile wings (aIF2α–D12) and main domain of aIF2β are anchored[3, 4] . The structure of the rigid core domain is shown on the center. The structure corresponds to a composite model made of Ss-aIF2γ (PDB entry 4RD4[5]) bound to α3 and the N-ter helix of β as observed in PDB entry 3V11[6]. aIF2α-D12 is at the upper left, and the core of aIF2β is at the upper right. The subunits are colored as follows: α in cyan, β in blue and γ in green, with its switch regions in pink.

(b) Superimposition of the crystal structure of the TC on the structure of the TC observed in IC0. The γ subunits of the two TC are superimposed. The TC from the crystal structure (PDB entry 3V11) is in dark blue. The TC from IC0 is colored as in Fig. 1a with γ in green, α in cyan, the N-terminal helix of β in blue and the tRNA in yellow. The view shows very good agreement of the two structures. Only aIF2α–D12 domains are slightly rotated from their positions seen in the crystal structure, accompanied by a minor movement of the initiator tRNA position.

(c) Structure of aIF1 from *Methanocaldococcus jannaschii* from PDB entry 4MO0.

(d) Structure of aIF1A from *Pyrococcus abyssi* from PDB entry 4MNO.

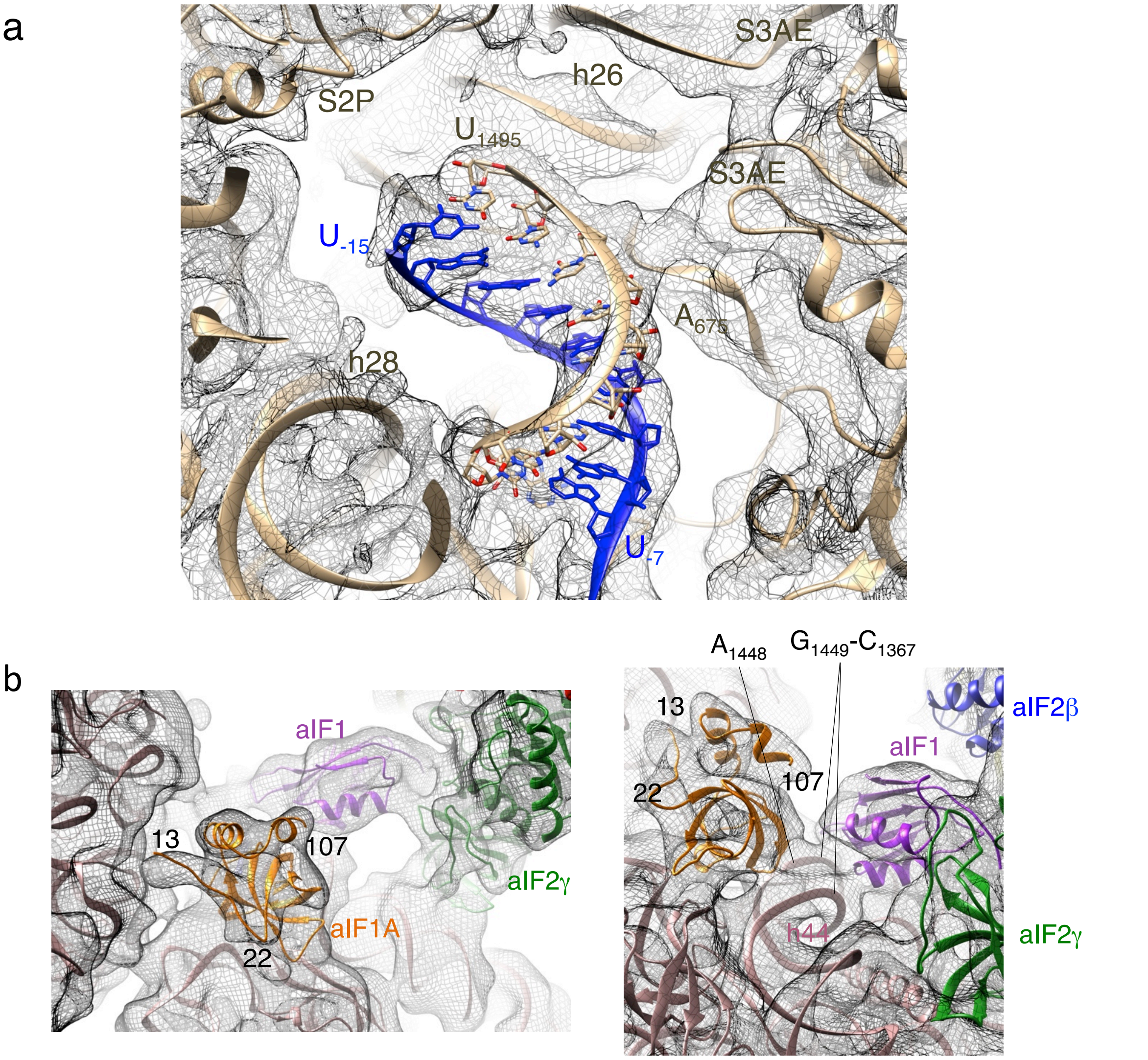

Supplementary Figure 4: Local fit of mRNA, aIF1 and aIF1A into IC0-P_{REMOTE} map.
(a) Region of the Shine-Dalgarno sequence of the mRNA base-paired with the rRNA as observed in the IC0-P_{REMOTE} electron density map filtered at 5.34 Å resolution.
(b) Fitting of Pa-aIF1A (PDB entry 4MNO) and Mj-aIF1 (PDB entry 4MO0) in IC0-P_{REMOTE} is shown in two different orientations. The cryo-EM map is filtered to 8.5 Å resolution.

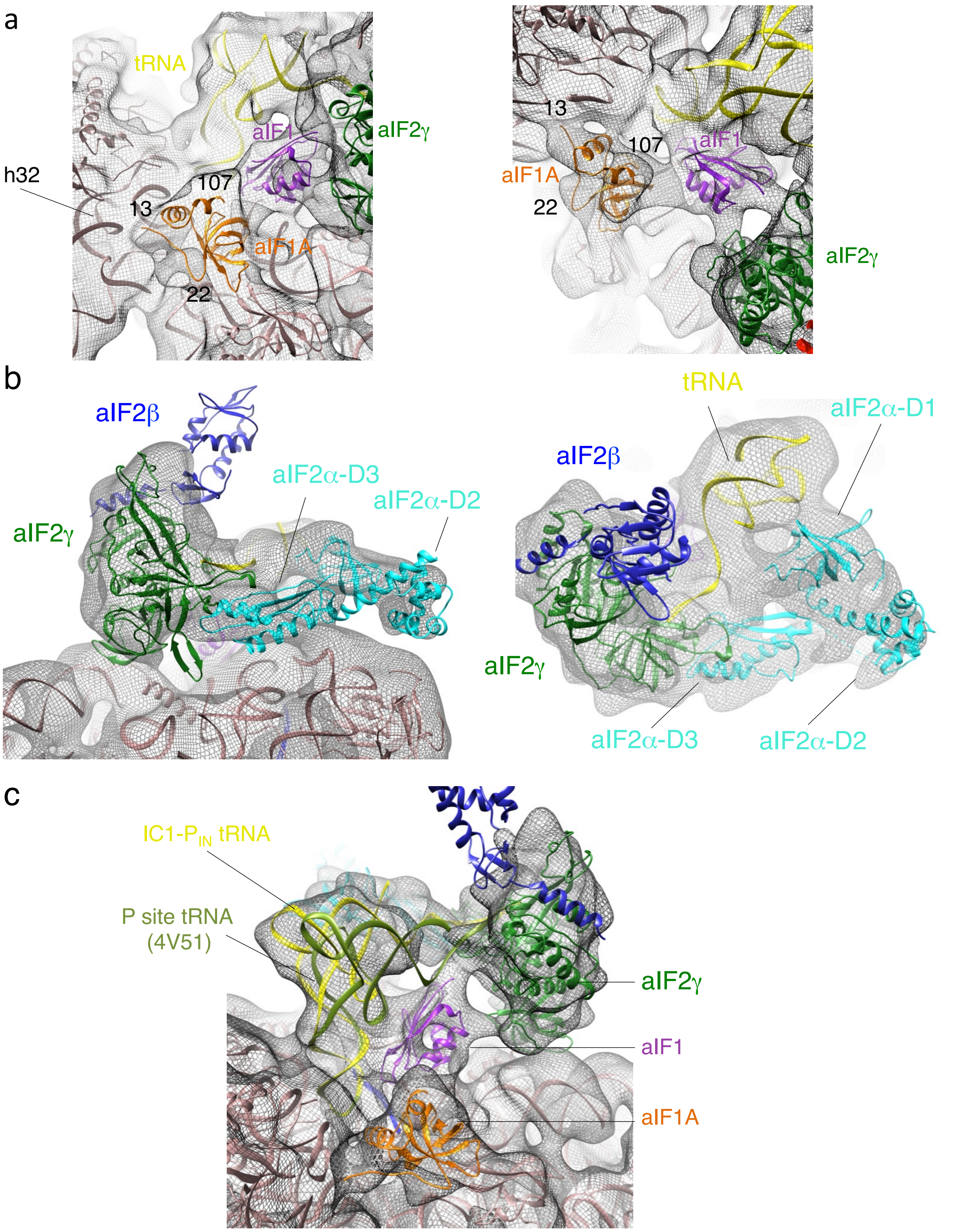

Supplementary Figure 5: Fitting of factors into IC1-P_{IN} map.
(a) Fitting of Pa-aIF1A (PDB entry 4MNO) and Mj-aIF1 (PDB entry 4MO0) in IC1-P_{IN}. The cryo-EM map is filtered to 12 Å resolution.
(b) Fitting of the ternary complex into IC1-P_{IN} map.
(c) Fitting of initiator tRNA (yellow) within IC1-P_{IN} map. The view shows that the structure of the P site initiator tRNA as observed in PDB entry 4V51[7] (light green) could not be readily rigid-body fitted in the electron density. A bending of the tRNA molecule was necessary to account for the density showing simultaneous binding of the initiator tRNA with the mRNA and aIF2.

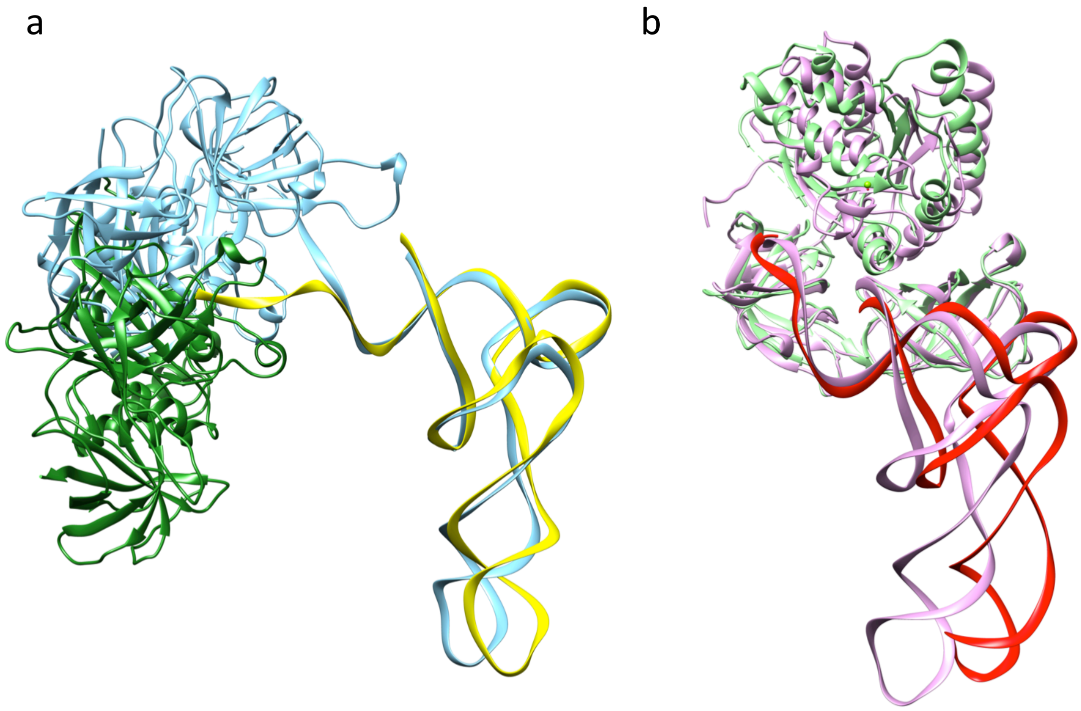

Supplementary Figure 6: Distortion of the tRNA in the aIF2 TC and in the EF-Tu TC.
(a) The free aIF2 TC structure (light blue, PDB entry 3V11[6]) and the structure of the TC in IC1-P_{IN} (tRNA in yellow and aIF2γ in green) are compared after superimposition of the tRNA acceptor stems. Only aIF2γ and the tRNA are shown.
(b) The free EF-Tu TC structure (purple, PDB entry 1TTT[8]) and the structure of the TC bound to the 30S subunit (tRNA in red and EF-Tu in light green, PDB entry 4V5G[9] are compared after superimposition of domains II and III of EF-Tu).
The figure illustrates that in both cases, tRNA accommodation results in distortion of the ternary complex as well as of the tRNA itself.

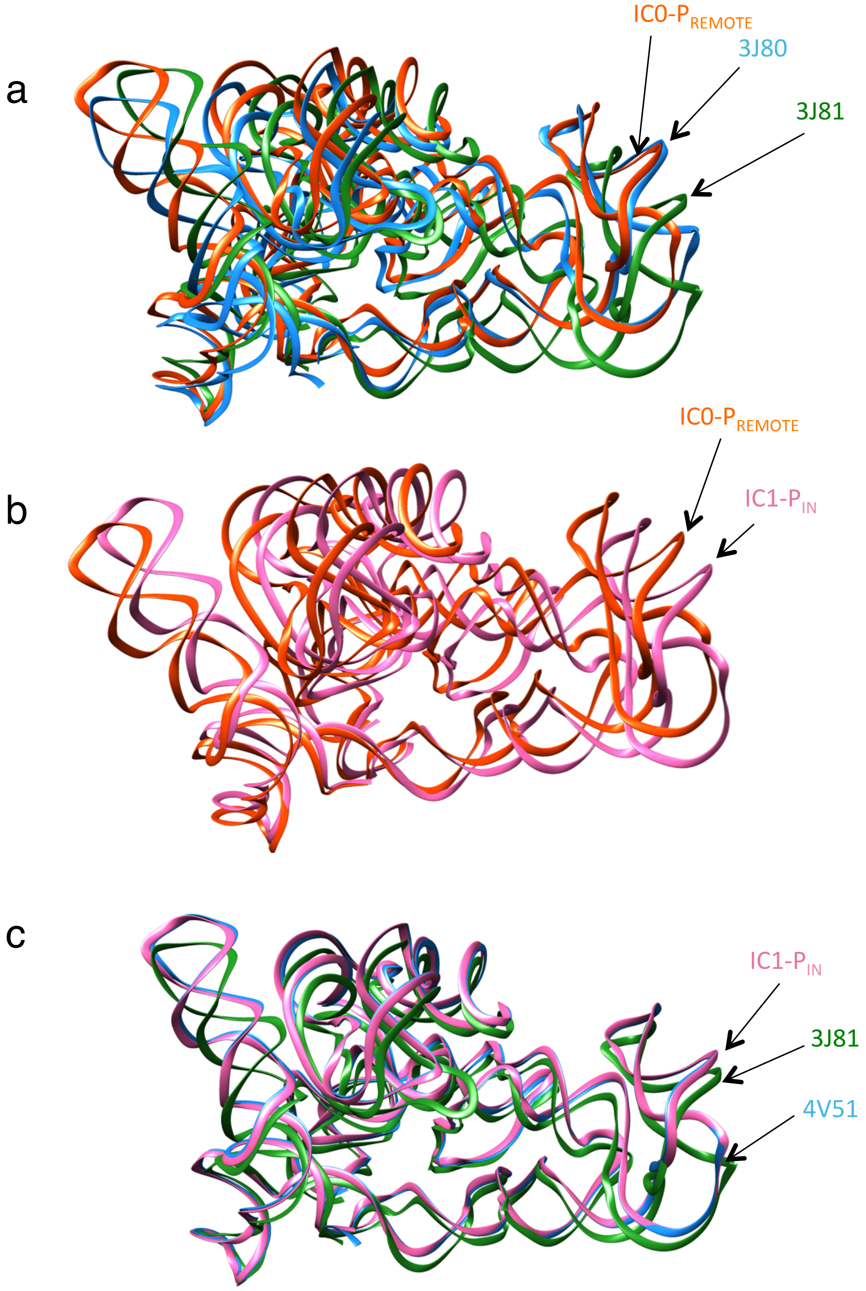

Supplementary Figure 7: Head movement upon transition from IC0-P$_{REMOTE}$ to IC1-P$_{IN}$.
Each panel compares position of the heads of the small ribosomal subunits after superimposition of the bodies.
(a) IC0-P$_{REMOTE}$ is compared to PDB entry 3J80 (40S:eIF1:eIF1A) and PDB entry 3J81 (py48S PIC)[10].
(b) IC0-P$_{REMOTE}$ is compared to IC1-P$_{IN}$.
(c) IC1-P$_{IN}$ is compared to PDB entry 3J81 (py48S PIC) and to PDB entry 4V51 (70S:mRNA:tRNA:paromomycin complex)[7].
The figure shows that the orientation of the head in IC0-P$_{REMOTE}$ is similar to that in 40S:eIF1:eIF1A whereas that in IC1-P$_{IN}$ resembles those in py48S PIC and 70S:tRNA complex.

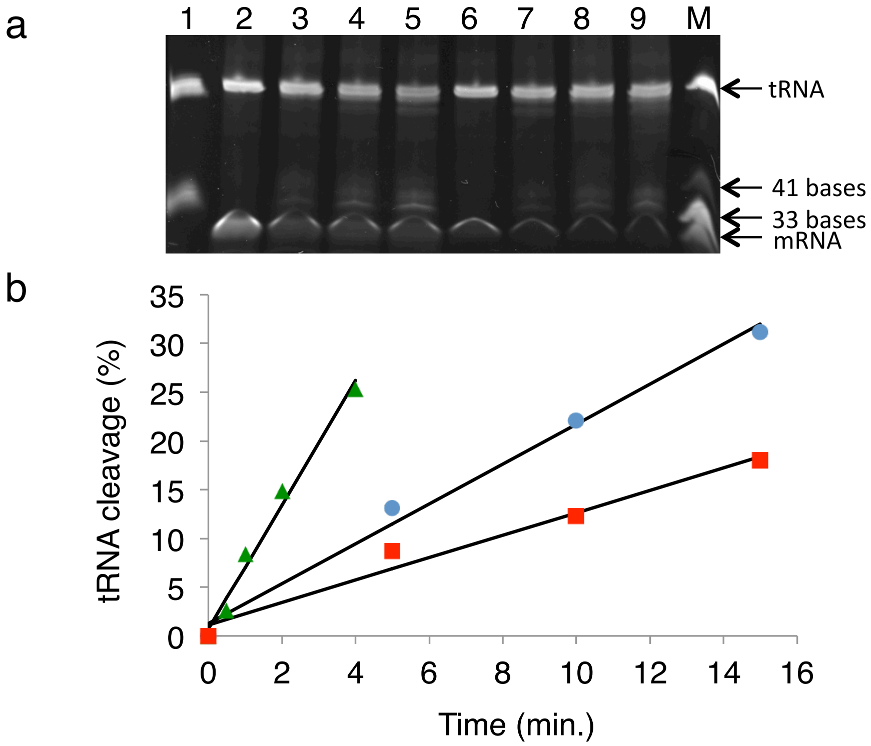

Supplementary Figure 8: Use of S1 nuclease as a probe of tRNA anticodon loop accessibility.
(a) Initiation complexes were assembled and submitted to S1 nuclease probing as described in Methods. The first complex was made with all initiation factors and CAU mRNA (lanes 2 to 5, incubation times of 0, 5, 10 and 15 min) in order to favor the P_{REMOTE} position of the initiator tRNA. The second complex was made without aIF1 and with an AUG mRNA (lanes 6 to 9, incubation times of 0, 5, 10 and 15 min) in order to favor the P_{IN} position of the initiator tRNA. Lane 1 was loaded with 100 ng of initiator tRNA submitted to S1 digestion with 1 unit of enzyme during 10 min at 51°C, pH 4.5. Lane M corresponds to a molecular weight marker containing intact tRNA (77 nucleotides), a 41-base DNA oligonucleotide, a 33-base DNA oligonucleotide and the CAU mRNA (26 nucleotides).
(b) Intensity of the intact tRNA band and of the cleavage bands were measured using ImageJ[11] and the percentage of cleavage was plotted as a function of incubation time. Blue circles are for complex 1 and red squares for complex 2. Straight lines show the linear regressions. The experiment was repeated three times, independently. Cleavage rates were 2.1 ± 0.07% per min and 1.2 ± 0.08% per min for complex 1 and 2, respectively (mean ± s.d from the three experiments). Cleavage rate of free Met-tRNA in the same conditions was 6.4% per min (green triangles). This experiment shows increased anticodon loop accessibility in complex 1 thereby supporting the existence of the P_{REMOTE} conformation in which the tRNA anticodon loop is accessible to S1 nuclease.

	IC0-P_{remote}	IC1-P_{IN}
**Data collection / processing**		
Microscope	Krios	
Voltage (kV)	300	
Defocus range (µm)	-0.5 – -2.8	
Exposure time per image (s)	1 (16 frames)	
Dose rate per image (e^{-} $Å^{-2}$)	19 (frames 2-7)	
Pixel size (Å)	1.12	
Number of images used in processing	2154	
Particles processed	192,025	
Particles refined	12,558	5,499
Resolution (unmasked, Å)	9.5	12.6
Resolution (masked, Å)	5.34	7.5
Map sharpening B-factor ($Å^2$)	-132	-208
**Refinement**		
Cell dimensions		
a = b = c (Å)	365,4	n. a.
α = β = γ (°)	90	n. a.
Resolution (Å)	5.8	n. a.
Bad bonds proteins (%)	0.07	n. a.
Bad angles proteins (%)	0.14	n. a.
Bad bonds RNA (%)	0.06	n. a.
Bad angles RNA (%)	0.95	n. a.
Good RNA backbone conformations (%)	76.06	n. a.
Correlation coefficient (model vs map,%)	82.1 (5.34 Å resolution)	88 (7.5 Å resolution)
Average FSC	0.56	n. a.

Supplementary Table 1: Data processing and refinement statistics. Values for model geometry were calculated with MolProbity[12].

SUPPLEMENTARY REFERENCES

1. Pettersen EF*, et al.* UCSF Chimera--a visualization system for exploratory research and analysis. *J Comput Chem* **25**, 1605-1612 (2004).

2. Schrodinger, LLC. The PyMOL Molecular Graphics System, Version 1.3r1. (2010).

3. Yatime L, Mechulam Y, Blanquet S, Schmitt E. Structure of an archaeal heterotrimeric initiation factor 2 reveals a nucleotide state between the GTP and the GDP states. *Proc Natl Acad Sci U S A* **104**, 18445-18450 (2007).

4. Stolboushkina E*, et al.* Crystal structure of the intact archaeal translation initiation factor 2 demonstrates very high conformational flexibility in the alpha- and beta-subunits. *J Mol Biol* **382**, 680-691 (2008).

5. Dubiez E, Aleksandrov A, Lazennec-Schurdevin C, Mechulam Y, Schmitt E. Identification of a second GTP-bound magnesium ion in archaeal initiation factor 2. *Nucleic Acids Res* **43**, 2946-2957 (2015).

6. Schmitt E*, et al.* Structure of the ternary initiation complex aIF2-GDPNP-methionylated initiator tRNA. *Nat Struct Mol Biol* **19**, 450-454 (2012).

7. Selmer M, *et al.* Structure of the 70S ribosome complexed with mRNA and tRNA. *Science* **313**, 1935-1942 (2006).

8. Nissen P, *et al.* Crystal structure of the ternary complex of Phe-tRNA^{Phe}, EF-Tu, and a GTP analog. *Science* **270**, 1464-1472 (1995).

9. Schmeing TM, *et al.* The crystal structure of the ribosome bound to EF-Tu and aminoacyl-tRNA. *Science* **326**, 688-694 (2009).

10. Hussain T, *et al.* Structural changes enable start codon recognition by the eukaryotic translation initiation complex. *Cell* **159**, 597-607 (2014).

11. Schneider CA, Rasband WS, Eliceiri KW. "NIH Image to ImageJ: 25 years of image analysis". *Nature Methods* **9**, 671-675 (2012).

12. Chen VB, *et al.* MolProbity: all-atom structure validation for macromolecular crystallography. *Acta Crystallogr D Biol Crystallogr* **66**, 12-21 (2010).
